# Multi‐target fluorescence staining of bacteria smears enables rapid machine learning‐assisted species classification

**DOI:** 10.1002/mlf2.70076

**Published:** 2026-04-16

**Authors:** Maxence Galvan, Michael Fujarski, Can Beslendi, Frieder Schaumburg, Julian Varghese, Johannes Liesche

**Affiliations:** ^1^ Institute of Medical Microbiology University Hospital Münster Münster Germany; ^2^ Institute of Medical Informatics University of Münster Münster Germany; ^3^ Institute of Medical Informatics Otto‐von‐Guericke University Magdeburg Magdeburg Germany; ^4^ Institute of Biology University of Graz Graz Austria

**Keywords:** artificial intelligence (AI), bloodstream infection, fluorescence microscopy, rapid diagnostics, resource‐limited settings

## Abstract

Rapid identification of bacterial species from patient samples is crucial for clinical decision‐making. In severe infections, such as bloodstream infections, the early start of an effective treatment is directly associated with reduced mortality rates. Current rapid species identification methods, such as matrix‐assisted laser desorption ionization time‐of‐flight mass spectrometry (MALDI‐TOF MS) or multiplex PCR, require specialized hardware and extensive technical support that prevents application in resource‐limited settings. Here, we present a staining and imaging procedure for bacterial smears using fluorescent dyes directed against intracellular structures and cell wall components. Data on relevant features were extracted from segmented images and used to train a machine learning (ML) model for species classification. The method was tested on clinical isolates from 126 patients. For the seven most common bacteria, the classification performance, indicated by area under the receiver operating characteristic (ROC) curve, ranged from 0.8 (*Klebsiella pneumoniae*) to 1 (*Pseudomonas aeruginosa*). Species that were not part of the training dataset, were reliably classified as unknown species. These results hold promise for the identification of further species, particularly *Enterobacterales*, and clinical application.

## Impact statement

This study introduces a cost‐effective method for bacterial species identification using fluorescent dyes and machine learning, reducing reliance on expensive equipment and specialized expertise. Tested on clinical samples from 126 patients, the technique achieved high accuracy for common bacteria like *Pseudomonas aeruginosa* and reliably flagged unknown species. Its simplicity and adaptability make it ideal for resource‐limited settings, offering faster diagnostic results to inform life‐saving treatments, especially for severe infections like bloodstream infections. This innovation has the potential to improve global healthcare by expanding access to advanced microbiological diagnostics and combating antibiotic resistance.

## INTRODUCTION

The microbiological diagnosis of infections is time‐sensitive, as mortality in severe infections, especially bloodstream infections, increases if an appropriate antimicrobial therapy is delayed^1^. In routine diagnostics, bacterial species identification is performed prior to the species‐specific antimicrobial susceptibility test (AST). Most laboratories incubate bacteria from positive blood cultures on solid media for 2 to 4 h^2^, before subjecting them to matrix‐assisted laser desorption ionization time‐of‐flight mass spectrometry (MALDI‐TOF MS), followed by mass spectra comparison with a fingerprints database for species identification^3^. MALDI‐TOF MS requires sophisticated hardware, technical support, regular maintenance, and adequate information technology infrastructure. These requirements limit its application in laboratories in low‐ and middle‐income countries^4^. In resource‐limited settings, culture‐based bacterial species identification using colony morphology, Gram staining, and biochemical test panels is performed instead. This typically yields results only after up to 48 h^5^. The delayed start of treatment due to slow species identification (if microbiological laboratories are available at all) could contribute to the high incidence of sepsis in sub‐Saharan Africa, the most severe form of bloodstream infection^6^, ^7^, ^8^. This highlights the need for rapid identification tools that meet the needs of laboratories with limited resources.

Staining of bacteria (e.g., Gram staining) has long been used for classification of pathogens^9^, ^10^. However, staining for species identification independent of other tests is currently limited to fluorescence *in situ* hybridization (FISH) with rRNA‐targeted oligonucleotide probes. FISH is generally not used for species identification in blood culture isolates in routine laboratories, as testing a library of probes directed against all potential pathogens would not be cost‐efficient^11^. Despite the vast pool of small organic fluorescent dyes^12^, no dyes or dye combinations have been found to identify medically relevant species with appropriate reliability^13^. One major issue is the dye specificity. While numerous potential species‐specific target features exist, even between closely related species^14^, ^15^, no single dye is specific enough for the identification of a broad range of species. The possibility of combining the information from different fluorescence stainings has received little attention, presumably because the limited spatial and spectral resolving power of common laboratory microscopes makes it difficult to reliably compare the relatively small differences in the staining pattern or fluorescence intensity across samples^16^. This challenge can be addressed by leveraging machine learning (ML) models, which offer greater precision and efficiency compared to traditional manual review and conventional image analysis methods^17^.

In this study, we tested if species identification in bacterial culture smears could be achieved with a combination of fluorescence stains and ML‐aided image analysis. The results could provide a basis for the development of simple and robust species identification methods for resource‐limited settings.

## RESULTS

### Selection of candidate dyes

**Figure 1:**
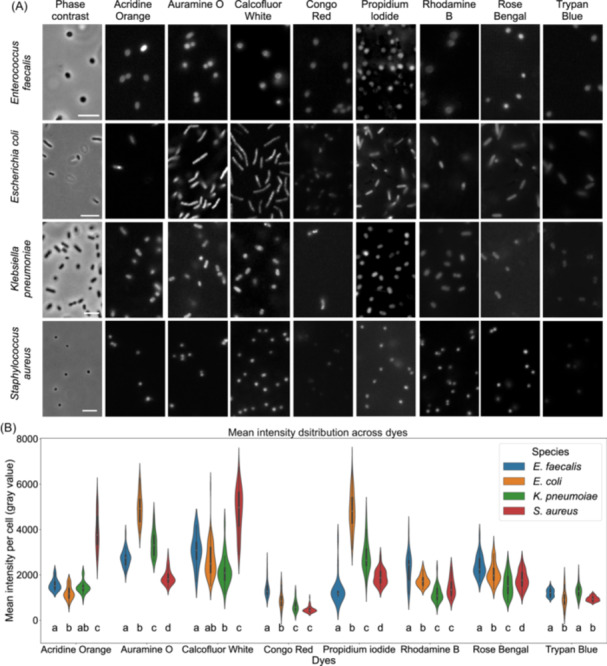
Selection of fluorescent dyes. (A) Representative images of cells in bacterial smears stained with the indicated fluorescent dyes. Scale bar, 5 µm. (B) Mean cellular fluorescence intensity of the eight dyes for *Enterococcus faecalis*, *Escherichia coli*, *Klebsiella pneumoniae,* and *Staphylcoccus aureus*. Detailed information on the dyes is provided in Table S1. Statistical analysis was performed using one‐way analysis of variance (ANOVA), followed by Tukey's HSD post‐hoc test. Groups not sharing the same lowercase letter differ significantly (*p* < 0.05). *n* = 30 cells from three biological replicates.

We assessed differences in fluorescence‐augmented morphometry of bacterial smears for species identification. This is a morphometric classification (e.g., perimeter, axis length, and solidity) augmented by general‐purpose fluorescence between stained bacterial smears. Accordingly, the first step was to test which fluorescent dyes could stain common bacteria with appreciable differences in intensity across species. Eight dyes provided images with high signal‐to‐noise ratio and minimal background staining on our wide‐field fluorescent microscope (Figure 1A). These were Acridine Orange, Auramine O, Calcofluor White, Congo Red, Propidium Iodide, Rhodamine B, Rose Bengal, and Trypan Blue. Details on their biological targets and relevant references are provided in Table S1.

The mean intensities of dyes were compared across four clinically relevant bacteria species: *Enterococcus faecalis*, *Escherichia coli*, *Klebsiella pneumoniae*, and *Staphylococcus aureus* (Figure 1B). Tukey's post‐hoc tests indicated that Auramine O and Propidium Iodide showed the strongest and most consistent species‐specific discrimination, with all pairwise comparisons significant (Figure 1B). Acridine Orange and Calcofluor White also demonstrated strong separation, particularly distinguishing *S. aureus* from other species. In contrast, Congo Red, Rhodamine B, and Rose Bengal showed moderate discriminatory ability, while Trypan Blue showed the weakest performance, with relatively small intensity differences and several non‐significant pairwise comparisons (Figure 1B). Besides Auramine O and Propidium Iodide, Acridine Orange was chosen to generate the image data for the following ML‐based analysis, as it showed lower within‐species variability than Calcofluor White.

### Generation of data for ML analysis

To design and optimize the ML model, we selected the 10 most common species found in bloodstream infections at the Münster University Hospital^18^: *Enterococcus faecium* (number of isolates: 12), *Staphylococcus epidermidis* (14), *S. aureus* (16), *E. faecalis* (10), *E. coli* (15), *Klebsiella oxytoca* (11), *Enterobacter cloacae* complex (11), *Proteus mirabilis* (12), *K. pneumoniae* (13), and *Pseudomonas aeruginosa* (12). For MALDI‐TOF MS analysis, each isolate was cultured on solid media plates (e.g., Columbia blood agar). Bacteria for microscopic analysis of smears were picked from the same plates on the same day for the MALDI‐TOF MS analysis; the results served as a reference. Bacterial smears on microscope slides were stained with Acridine Orange, Auramine O, and Propidium Iodide. In total, 3780 images of smears from 126 isolates were recorded. Images typically showed between 20 and 300 cells, and 926,134 individual cells were analyzed in total. Details on numbers of analyzed cells per species are provided in Figure S1.

To check if dyes influence cell structure, we assessed whether structural features of the bacteria were consistent across different dyes. We compared the distributions of axis lengths (minor and major), cell area, and eccentricity across species and dyes (Figure S2). Overall, these parameters were consistent between dyes, with low effect sizes (*η*² typically <0.15) indicating minimal dye‐related variation. For example, eccentricity values (Figure S2D) showed near‐identical distributions across dyes, confirming that bacteria shape appearance was dye‐independent. Similar observations were made for cell size‐related parameters such as area and major/minor axis lengths (Figure S2A–C), with the largest effect, although still relatively small, observed in *K. pneumoniae* minor axis length (*η*² = 0.23).

A pre‐trained image segmentation algorithm (Omnipose^19^) was adapted to mark the outline of single cells on each image (see Materials and Methods section for details). Information on fluorescence intensity and cell shape parameters was extracted for each cell using scikit‐image.^20^ Many of these parameters were correlated with each other (Figure S3). Feature reduction was carried out to reduce irrelevant data by restricting the analysis to 11 parameters with limited cross‐correlation, which were area, eccentricity, major/minor axis length, perimeter, solidity, angular second moment (ASM) of the co‐occurrence matrix, contrast, correlation, dissimilarity, homogeneity, and mean intensity. The information of whether the bacteria on the culture plate are Gram‐positive or Gram‐negative was manually added as a binary variable to the image‐derived data for each smear sample (“Gram staining”).

**Figure 2:**
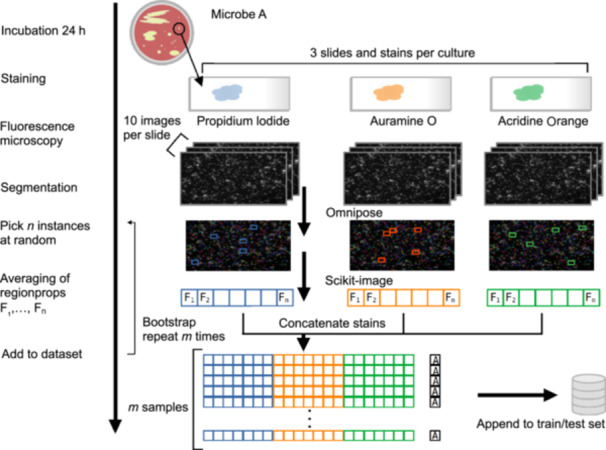
Workflow for the generation of a machine learning (ML) dataset. Three bacterial smears from each isolate were stained, each with one of the three dyes. Images were segmented by Omnipose and features were quantified for each cell via scikit‐image. The mean values of the features from 25 cells per dye per isolate were randomly combined into sample sets, of which 1000 were generated. Sample sets were combined in the train/test dataset.

After staining the three smears from each isolate with the different dyes, 10 images were recorded for each smear (Figure 2), resulting in 30 images per isolate. After segmenting and measuring the relevant features for each cell on each image, data from 25 cells were selected randomly for each dye, and their mean feature values were collected as one sample set. This was repeated 1000 times (bootstrapping, Figure 2), resulting in 1000 sample sets for each isolate. All sample sets were collected in the train/test dataset.

The use of the mean feature values from a random set of *n* segmentations instead of the feature values for each individual segmentation was found to result in a significant improvement in classification performance (Figure S4). Manual review indicated that this improvement was due to a reduction of the influence of unusual microbial morphology or segmentation errors. The benefit was larger than the cost of losing some information on cellular heterogeneity. The train/test dataset was divided at an 80:20 ratio with a stratified and grouped five‐fold cross‐validation to ensure that the testing set is truly independent from the training set. We used the CatBoost ML method on these data.

### Classification of bacteria included in the training set

Our classification model was trained on seven of the 10 available species (*S. epidermidis*, *S. aureus*, *E. faecalis*, *E. coli*, *K. oxytoca*, *K. pneumoniae*, and *P. aeruginosa*). Data from the three additional species (*E. faecium*, *P. mirabilis*, and *E. cloacae*) were used to test the model performance on unknown species, as described in the following section.

**Figure 3:**
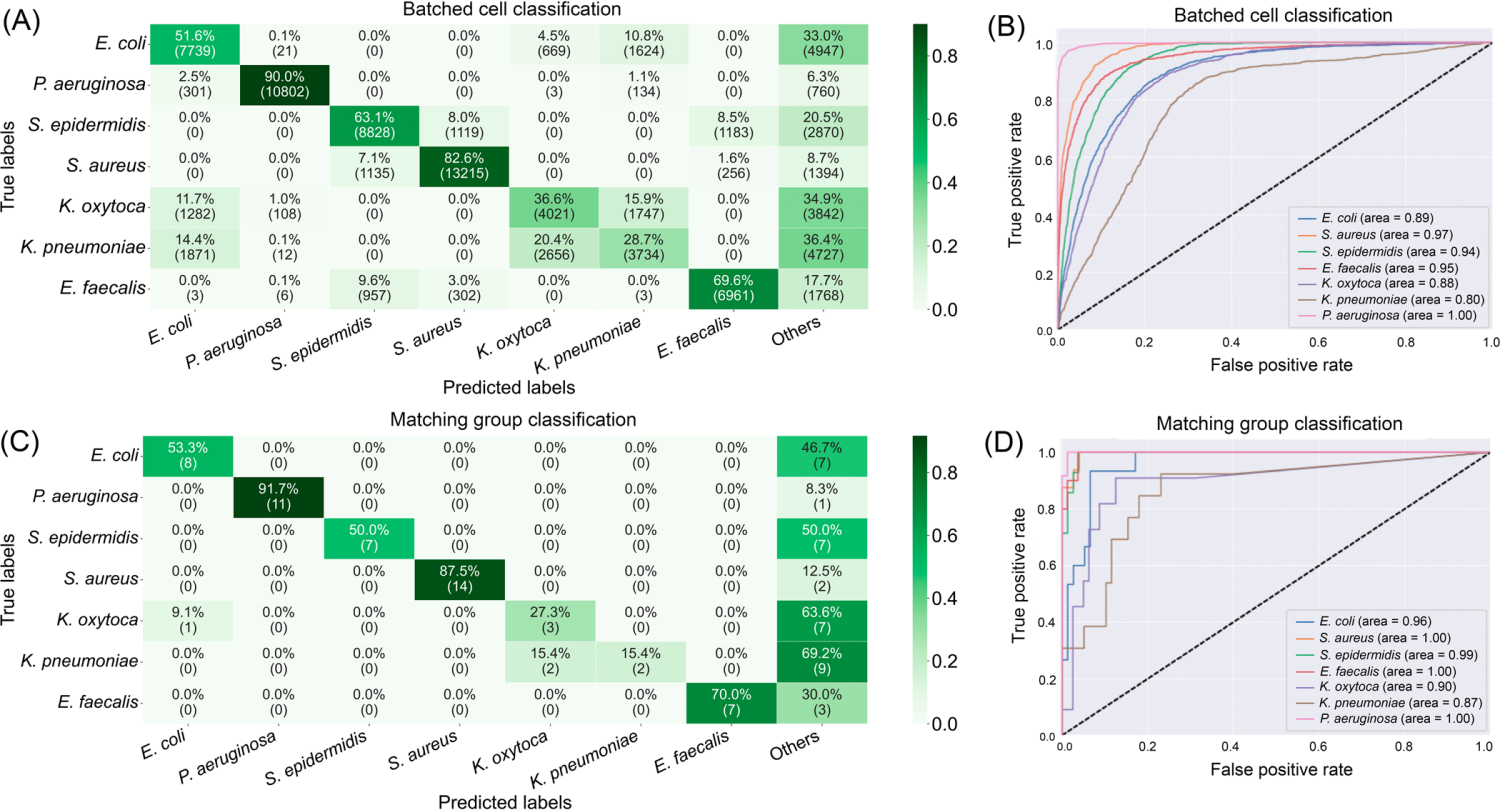
Performance of the classification model. The model treated each batch of 25 randomly selected cells separately (A, B) or operated on the majority voting results of 1000 batched cell sample sets belonging to one isolate (C, D). (A, C) Confusion matrices of the classification model indicating the number of correctly classified and misclassified samples. (B, D) Receiver operating characteristic (ROC) curves indicating the performance of the batched cell classification (B) or matching group classification (D) for the different bacteria as the area under the curve after five‐fold cross‐validation. “Others” refers to instances that could not be classified as one of the species included in the training set with a prediction accuracy above 75%. Confusion matrices of results with thresholds at 50% and 0% are provided in Figure S5. ROC curves indicating classification performance for each species at different numbers of cross‐validation are provided in Figure S6.

We analyzed the performance of the model on the data produced from the mean feature values from 25 randomly selected cells (“batched cells”) and observed that the model correctly classified most included species (*E. coli*, *P. aeruginosa*, *S. epidermidis*, and *S. aureus, E. faecalis*, “batched cell classification”, Figure 3A). Misclassifications occurred mostly between rod‐shaped species such as *E. coli*, *K. oxytoca*, and *K. pneumoniae*, with misclassifications ranging from 19% to 38% of sample sets (Figure 3A). Similarly, but less frequently, a misclassification of species belonging to cocci such as *S. epidermidis* and *S. aureus* was observed (16% and 9%, respectively). In addition, some species were frequently misclassified as “others,” meaning that a sample set could not be assigned to a single species with a prediction accuracy above 75%. These were *E. coli* (31%), *S. epidermidis* (21%), *K. oxytoca* (35%), and *K. pneumoniae* (36%, Figure 3A). Lowering this threshold to 50% or 0% led to a higher share of correctly classified cells, but also increased the number of false‐positives, that is, cells classified as one of the other species (Figure S5A,B).

To evaluate the performance of our batched cell classification model, we calculated the receiver operating characteristic (ROC) curves and corresponding area under the curve (AUC) values for all seven species (Figure 3B). The AUC for all species exceeded 0.80. The AUC was the highest for *P. aeruginosa* (1.00), followed by *S. aureus* (0.97), *E. faecalis* (0.95), and *S. epidermidis* (0.94, Figure 3B). The lowest AUC value was observed for *K. pneumoniae* (0.80). Thus, the model demonstrates high AUC values for the majority of species, which indicates an overall robust performance.

This method is intended for use in classifying single isolates. To better represent this scenario, we trained and tested the ML model on the majority votes of each of the 1000 sample sets per isolate. This led to increased model performance and classification specificity (Figure 3C,D). Misclassification was eliminated for all but the *Klebsiella* species. However, the share of classifications of species as “others” increased, sometimes even reaching more than 50% (Figure 3C). This value depends on the threshold for the “others” labeling. Here, any value less than 75% of cells recognized as belonging to one specific species led to classification as “others.” As seen above for the batched cell classification, the share of correctly classified isolates increased with a lower threshold, but so did the number of misclassifications (Figure S5C,D).

### Classification of bacteria not included in the training set

**Figure 4:**
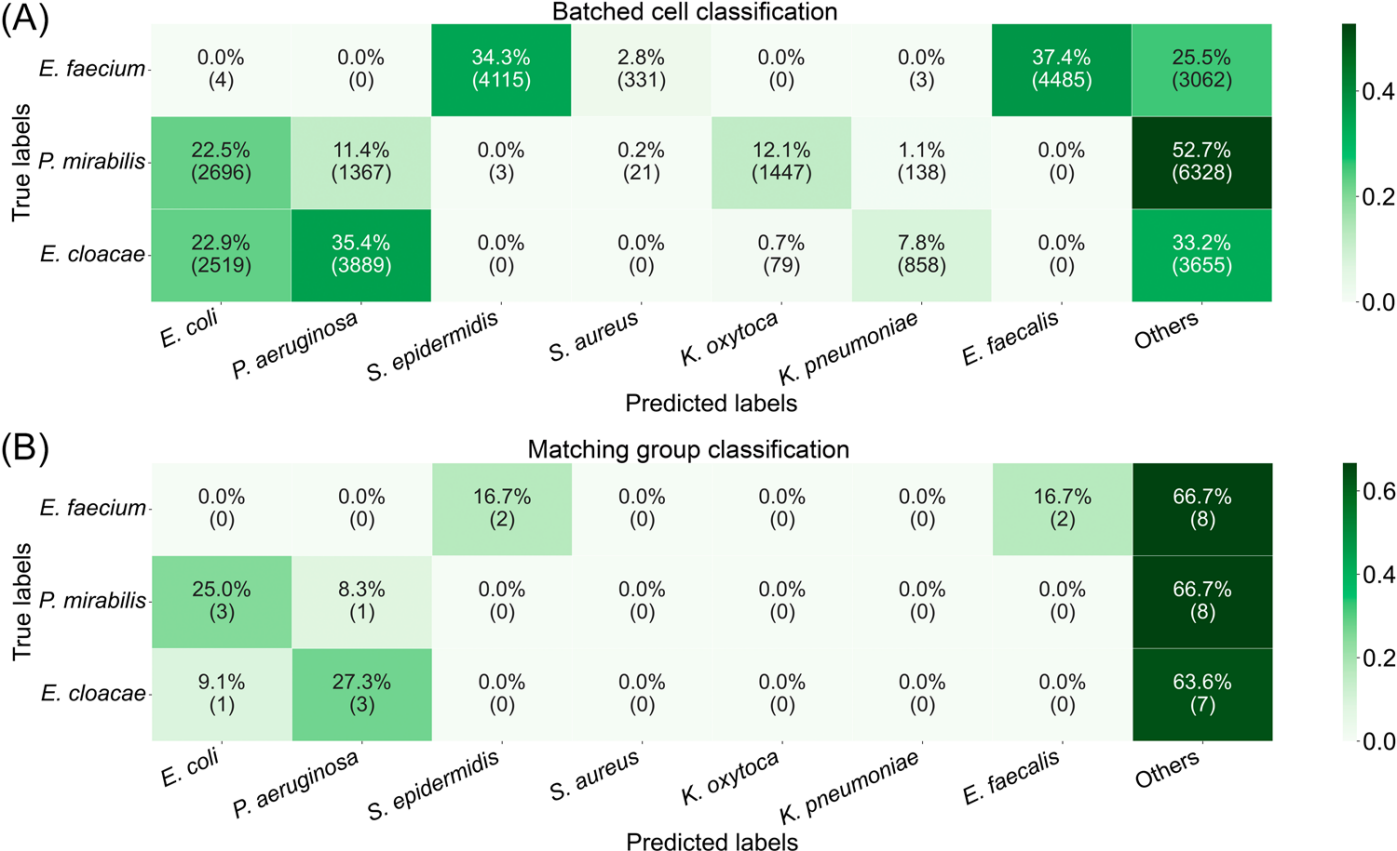
Confusion matrix showing the performance of the classification model in recognizing unknown species. Either batched cell classifications (A) or majority of votes matching group classification on the 1000 batched cell sample sets derived from the same original isolate (B) were considered. Confusion matrices of results with threshold at 50% are provided in Figure S7.

The three species, *E. faecium*, *P. mirabilis*, and *E. cloacae*, which were not included in the training dataset, were used to evaluate the model's ability to handle unknown bacterial species. In an ideal scenario, the model should classify these batched cell samples as “others”. As illustrated in Figure 4A, the correlation matrix demonstrates that the model correctly classified 52.7% of all *P. mirabilis* samples as “others,” 33.2% of *E. cloacae* samples, and 25.5% of *E. faecium* samples. Frequent misclassification occurs with species that are closely related or have a similar structure. The model misclassified *E. faecium* in 71% cases as *E. faecalis* (37.4%) or as *S. epidermidis* (34.3%). Additionally, *E. cloacae* was predominantly misclassified as either *P. aeruginosa* (35.4%) or *E. coli* (22.9%). Among the species, *P. mirabilis* showed the lowest misclassification rate (22.5% *E. coli*, 12.1% *K. oxytoca*, and 11.4% *P. aeruginosa*).

Like the performance differences seen in the classification of batched cells compared to cohesive sample groups, a significant improvement in specificity for labeling unknown samples was observed when the model classifies the sample group using majority voting (Figure 4B). *E. faecium* or *P. mirabilis* were correctly classified as “others” in 66.7% and *E. cloacae* 63.6% of the time. While the model maintains its misclassification pattern by misclassifying structurally similar microbes, the extent of misclassification was reduced.

The prediction accuracy threshold for classification as “others” was set to 75%. Reducing the classification threshold to 50% strongly increased the number of misclassified instances for all three species (Figure S7).

### Structural features contribute strongly toward prediction accuracy

**Figure 5:**
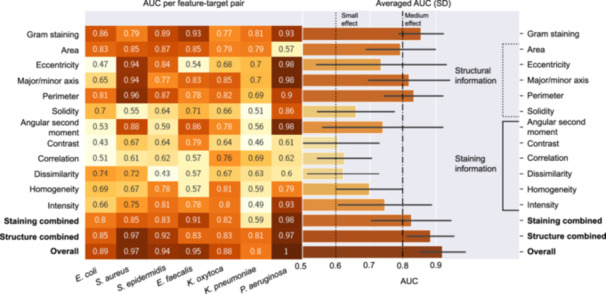
Importance of structural and staining features for the classification model accuracy. The effect of the permutation of individual features on model performance was tested. Conventional effect thresholds at 0.6 (small effect) and 0.8 (medium effect) were used to evaluate the importance of individual features according to the respective area under the curve (AUC) of the ROC. Error bars show standard deviations. Separate feature importance analyses for the three dyes are provided in Figure S8.

To further improve the classification model, it is important to ascertain the parameters on which the classification is most dependent. To evaluate the importance of the different parameters, we analyzed the influence of permutation of individual features on the model AUC (Figure 5). The stronger the influence of feature permutation on AUC values, the closer the resulting individual AUC values become to 1. Typically, individual AUC >0.6 indicates that leaving out this feature would significantly decrease the model's prediction accuracy. Our results show that the structural information has higher individual AUC values than the staining features (Figure 5). The features with the strongest effect on model performance are Gram staining, major/minor axis, and perimeter, with averaged individual AUC values above 0.8 (Figure 5). The staining features with the highest individual AUC values are the ASM of the co‐occurrence matrix and intensity, which displayed a moderate effect size (Figure 5). Some features, such as eccentricity, display high variability in AUC scores across different species (0.98 for *P. aeruginosa* and 0.47 for *E. coli*). Features with the lowest average AUC values are contrast, dissimilarity, and correlation, with AUC values below 0.8 for each species. However, with an average individual AUC of around 0.6, their inclusion remains reasonable. When feature importance was analyzed separately for Acridine Orange and Auramine O, a high similarity in relative importance and AUC values was observed (Figure S8).

## DISCUSSION

Fast reliable identification of bacteria in clinical samples is a critical factor in microbiological diagnostics^21^. Recently, optics‐based solutions for this task have come into focus, partly due to higher accessibility and lower costs of microscopy, spectroscopy, and microfluidics hardware^22^, ^23^. However, proposed methods, such as Raman spectroscopy, still feature a high level of technical complexity. Fluorescence‐based innovations have mostly focused on novel probes with high target specificity^13^, ^24^, which could be incompatible with cost efficiency requirements in low‐resource hospitals. In contrast, our protocol uses relatively inexpensive, widely available fluorescent dyes. We show that, in combination with ML‐assisted image analysis, staining with these dyes can be used for microbial species identification.

The different molecular targets of the dyes form the mechanistic basis of this approach, as they lead to species‐specific fluorescence‐augmented morphometry patterns when interacting with cell structures of the different species. The intensity of dyes, such as Auramine O and Calcofluor White, that bind to specific cell wall components is influenced by these components' abundance and accessibility. Intensity differences of dyes that bind to intracellular components, especially DNA, could mainly reflect differences in accessibility, and potentially also differences in pH and low‐specificity dye binding to various molecules. For example, Acridine Orange is likely to bind, besides nucleic acids, to anionic structures whose distribution and abundance can vary between bacterial species. Testing of a broader range of dyes and labels is likely to reveal more that could improve the ability to discriminate between species.

Our samples were the same as those used in the current best‐practice approach for the identification of pathogenic microorganisms in blood culture bottles, in which cultivation on solid media is followed by MALDI‐TOF MS analysis^3^. Since our approach is based on wide‐field fluorescence microscopy and the analysis can be run on a personal computer, the method could be more cost‐effective than MS analysis. Moreover, the use of nonspecialized hardware alleviates issues of maintenance that can limit the value of complex instruments in resource‐limited settings.

The AUC of the receiver‐operating characteristics, a common indicator of classification performance, varies between 0.8 and 1 for the different bacteria tested here. The prediction accuracy of our method could be increased, at least for some bacteria, by extending the training data (Figure S9). A larger sample size will presumably enable a more robust species classification by reducing noise sensitivity and the influence of differences in imaging conditions. Improvements could also be made by using additional dyes with different targets and suitable staining properties. Besides fluorescence‐augmented morphometry, information from other tests, for example, images of colony growth patterns of the plate cultures, could further improve the predictive performance. Gram staining, used here as a feature, might have a greater impact if it was used as an initial classification step before ML analysis. Moreover, the precision of our segmentation model could be enhanced to reduce the rate of errors in the segmentations. In our current approach, we utilized the same pre‐trained model from Omnipose on each dye instead of fine‐tuning with custom data to different shapes and sizes.

Despite these options for improvement, the similarity of some species means that it might be practically impossible to reduce the false‐positive rate to zero for all species. There is a risk for misclassification (e.g., *K. pneumoniae* vs. *K. oxytoca,* or *S. aureus* vs. *S. epidermidis*, Figure 3), particularly if the respective species was not part of the training dataset (*E. faecium* vs. *E. faecalis* and *S. epidermidis* or *E. cloacae* vs. *P. aeruginosa* and *E. coli*, Figure 4). These misclassifications can have a negative clinical impact, as wrong antimicrobial agents could be selected for treatment. While some of these misclassifications can be easily resolved by an affordable test such as the coagulase test to differentiate between *S. epidermidis* and *S. aureus* ^25^, or the oxidase test to differentiate *P. aeruginosa* from *Enterobacterales* ^26^, other misclassifications are more challenging and need to be addressed, mainly by larger training sets and/or additional dyes. Finally, it remains the responsibility of the microbiologist to check the results of species identification and AST for plausibility and expected AST phenotypes. Unexpected AST phenotypes should always prompt further efforts to verify the species.

Nevertheless, implementation of our method could still be of value to clinicians, particularly in hospitals without access to an instrument for MS analysis. Since the model yields information on the most likely candidates, it can simplify the follow‐up analysis with conventional tests.

One limitation of this study is the relatively low number of microorganisms that we trained the classification model for. While the 10 species represent the most common pathogenic microbes for bloodstream infections in a German hospital^18^, there are 30–50 species that are also clinically relevant (e.g., *Acinetobacter* spp., *Streptococcus* spp.)^27^. Our algorithm could be trained to detect these in the same way as for the species that were included. The extension of training data would also need to take regional differences of relevant species into account. To classify species that only rarely cause infections, samples for training would need to be collected over a longer period or from a larger number of hospitals.

In conclusion, our findings demonstrate that bacterial species identification based on fluorescence‐augmented morphometry classification has the potential to identify commonly encountered bacterial species in routine diagnostics. We therefore want to further develop this method for an intended use of first‐line species identification in regions with limited resources, where only a few ambiguous results need to be clarified by biochemical testing.

## MATERIALS AND METHODS

### Experimental model

We used bacterial colonies from clinical samples collected at Münster University Hospital.

### Culture

The 10 species most commonly found in blood cultures at our institution (*E. faecium*, *S. epidermidis*, *S. aureus*, *E. faecalis*, *E. coli*, *K. oxytoca*, *E. cloacae* complex, *P. mirabilis*, *K. pneumoniae*, and *P. aeruginosa*)^18^ were included in this study once they were detected in blood cultures (convenience sampling). No exclusion criteria were applied and strain variation was not evaluated. The culturing procedure followed Froböse et al.^2^ Briefly, 50 μl of blood cultures flagged as positive by a blood culture system (BD BACTEC FX; BD) was streaked out on Columbia blood agar plates (BD). Plates were incubated at 36 ± 1°C. After 6 h, MALDI‐TOF MS was carried out on single colonies for species identification. Bacterial smears for staining were prepared from single colonies after 22–26 h of incubation.

### MALDI‐TOF mass spectrometry

Species identification was performed on a MALDI‐TOF mass spectrometer (Biotyper Sirius; Bruker) using the MBT Compass IVD 4.2 database according to the manufacturer's recommendations. Microbial biomass from a single colony was transferred to a polished steel MALDI target using a sterile toothpick. Measurements were carried out after application of 1 μl of 70% formic acid and overlaying with α‐cyano‐4‐hydroxycinnamic acid.

### Staining and imaging

Stock solutions of the fluorescence dyes Acridine Orange, Auramine O, Congo Red, Rhodamine B, Trypan Blue (Carl Roth GmbH), Calcofluor White, Propidium Iodide, and Rose Bengal (Merck Millipore) were generated by diluting 10 mg in 10 ml of water, except for Acridine Orange (5 mg in 10 ml of water) and Propidium Iodide (0.5 mg in 10 ml of water). Working solutions were produced by a 1:10 dilution of an aliquot of the stock solutions. Calcofluor White (1 mg/ml Calcofluor White M2R, 0.5 mg/ml Evans blue) was purchased as the working solution.

Bacterial smears were produced by transferring single colonies from overnight cultures to glass slides using sterile inoculating loops. The bacteria from the tip of the loop were spread on a large cover glass (24 mm × 50 mm) by moving the tip over the cover glass in a wave form. Smears were dried for 30 s at room temperature before 15 µl of the staining solution was applied. The smear was covered with a small cover glass (22 mm × 22 mm). Smears stained with the different dyes (eight for initial tests and three for producing the test–train data set) were prepared from similar‐sized colonies of each isolate. Imaging of bacterial smears was performed within 5–15 min after application of staining solution on a Zeiss Axio Observer 7 fluorescence microscope using Zeiss ZEN blue (version 3.2.0; Carl Zeiss Microscopy GmbH). The microscope was equipped with an Axiocam 503 mono CCD camera, an HXP 120 V lighting unit, and a Plan‐Neofluar 100×/1.30NA Oil Ph3 objective (Carl Zeiss Microscopy GmbH), which provides a magnification of 100× at the camera. The camera has a physical pixel size of 4.54 µm and provides images of 1936 × 1460 pixels. Camera gain was left at the base level (“0”). For each staining solution, pre‐experiments were conducted to find optimal settings of exposure time and filters. These settings were then kept constant in all experiments. The filter combination used for Calcofluor White was an excitation filter with transmission up to 365 nm, a dichroic mirror with edge at 395 nm, and an emission filter with transmission of light at 420 to 470 nm. The filter combinations used for the dyes Acridine Orange and Auramine O were an excitation filter with transmission at 450 to 490 nm, a dichroic mirror with edge at 495 nm, and an emission filter with transmission of light at 500 to 550 nm. The filter combinations used for the dyes Congo Red, Rhodamine B, Propidium Iodide and Rose Bengal, and Trypan Blue were an excitation filter with transmission at 538 to 563 nm, a dichroic mirror with edge at 570 nm, and an emission filter with transmission of light at 570 to 640 nm. Exposure times ranged from 10 to 300 ms, as specified in Table S1.

After adjusting the focus to the cells closest to the objective lens, images were taken at positions where single cells were visible. Areas where cells were densely clustered were avoided.

### Instance segmentation

The acquired images were normalized using histogram equalization and segmented using the publicly available framework Omnipose^19^ (version 0.4.4). We used the pre‐trained model bact_fluor_omni. We reduced the flow_threshold from 0.4 to 0.1, which reduces the allowed discrepancy between predicted and actual flow field. Furthermore, we increased the mask_threshold to 1.0. This configuration enabled segmentation to include the well‐defined in‐focus cells, while ignoring low‐contrast cells that would lead to unreliable segmentation. Other parameters were used at default settings following the recommendation by the authors of Omnipose^19^. The quality was determined visually and statistically. All images were segmented using the same model parameters regardless of the dye, the species, or their assignment to the train or test set. The segmentations contained a 2‐dimensional array per image with a numeric identifier per pixel that associated pixels to specific instances. The masks and intensity images were used to extract local features for each microbe. Features were extracted in Python using the package scikit‐image (version 0.21.0). We used its regionprops method to calculate the relevant features, as described in the Results section. The full data are provided in Table S2.

### Quantification and statistical analysis

To perform a sanity check on the images and masks, we conducted a descriptive analysis using 10 images per sample and dye (Figure 2). To ensure comparability among different images with varying microbe densities, we averaged local features of microbes for each image separately. Bootstrapping was utilized for averaging by picking 25 random microbes per sample and 1000 evenly distributed samples per group of 10 images. We then compared the distributions of different feature means among the different stains. First, we compared the shape of the microbes by evaluating the circularity and eccentricity. Since the fluorochromes are not likely to alter the shape of the microbes, we expected consistent results among the dyes, most of which primarily target the cell wall. Next, we compared the average size and major axis. Lastly, we compared the image intensities for the different staining methods. While the first two categories of features were supposed to be consistent for the dyes selected, the intensity should have larger disparities in order to contain a predictive value.

We further conducted a correlation analysis on the available data. We calculated the point‐biserial correlation coefficient between the dichotomous microbe label and the continuous features. We considered a coefficient *r* ≥ 0.1 as a small effect, *r* ≥ 0.3 as a medium effect, and *r* ≥ 0.5 as a large effect^28^. Correlations with an adjusted *p* > 0.05 or coefficients *r* < 0.1 were considered as not significant and discarded. In order to reduce the number of features used during ML, we additionally evaluated the inter‐feature correlation. Since the underlying distributions did not pass the Kolmogorov–Smirnov test, we applied the nonparametric Spearman's rank correlation coefficient. Any *p* values were corrected using the Bonferroni correction. The threshold for significance was set to adjusted *p* < 0.05 for all tests.

### Machine learning

To avoid overfitting on confounding variables, that is, batch effects, we grouped the 10 images of each stain according to the isolate. Features were extracted per stain and the data concatenated for training and testing. The used features were reduced according to the inter‐correlation of the features (Figure S3). To ensure high reliability of the calculated features, we tested different batch sizes of cells and averaged the resulting values, with 25 cells per stain yielding reliable results. If not stated differently, the term batched cells refers to this averaging of 25 cells per image.

All available samples were randomly split into a training set and a testing set. To prevent an imbalanced dataset, the split was conducted using a group split based on segmentation count (i.e., number of cells) per species and dye. A train/test split of 80:20 was chosen according to the results of a stratified and grouped fivefold cross‐validation (Figure S6).

Due to the manageable number of features, we focused the model selection on three common algorithms: the kernel‐based support vector machine (SVM) and the two decision tree‐based algorithms Random Forest and CatBoost. CatBoost, in contrast to Random Forest, utilizes gradient boosting with symmetric decision tree generation as well as sophisticated handling of categorical data, resulting in improved performances with lower risk of overfitting. Optimizations of the models (Random forest, CatBoost and SVM) and model performances were conducted on the training set using a stratified cross validation in a one vs. rest approach. Model performance was evaluated according to prediction accuracy. The best model (CatBoost) was evaluated on the remaining test set to avoid the aforementioned overfitting.

The used metrics to evaluate the performance consist of precision, recall, and AUC. The metrics were recorded for two separate prediction tasks. First, we trained the model on a subset of the available species. The subset was then used to determine the predictive performance on a set of known species. Second, the remaining held‐out species were used to determine the specificity and false‐positive rate of the model if exposed to previously unknown samples.

### Feature importance analysis

Feature importance testing was conducted using SHapley Additive Explanations (SHAP) values. The models of the training step were evaluated using a permutation explainer on the hold‐out folds of the stratified cross‐validation. SHAP values were calculated for each feature separately, enabling the evaluation of each feature individually as well as combinations of features in semantically meaningful subgroups. Distributions of single and combined SHAP values were evaluated using the AUC values. Given the direct correspondence between AUC and Somers' D, a measure of association between two possibly dependent random variables, we expressed effect sizes in terms of AUC. Accordingly, we considered AUC values above 0.6 and 0.8 to represent small and medium effect sizes, respectively. Values above 0.9, not present here, would be considered a large effect size.

## AUTHOR CONTRIBUTIONS

**Maxence Galvan**: Data curation; formal analysis; investigation; methodology; software; validation; visualization; writing—original draft; writing—review and editing. **Michael Fujarski**: Formal analysis; methodology; software; validation; visualization; writing—review and editing. **Can Beslendi**: Investigation; methodology. **Frieder Schaumburg**: Conceptualization; funding acquisition; methodology; project administration; resources; supervision; writing—review and editing. **Julian Varghese**: Methodology; resources; supervision; writing—review and editing. **Johannes Liesche**: Conceptualization; data curation; formal analysis; funding acquisition; investigation; methodology; project administration; resources; supervision; visualization; writing—original draft; writing—review and editing.

## ETHICS STATEMENT

No patient‐related data were used in this study. Therefore, an ethical approval was not requested. Bacterial colonies used in this study were collected from clinical samples at Münster University Hospital, with no involvement of patient‐related data.

## CONFLICT OF INTERESTS

The authors declare no conflict of interests.

## Supporting information

suppl material revised‐2.

Table S2.

## Data Availability

The raw data set produced in the current study is provided in Table S2. The source code is available on GitLab at https://imigitlab.uni-muenster.de/published/microbe-identification. All data are made available with the publication, and the code is shared on GitHub to ensure transparency and reproducibility.
