# Supplementary material for: Multi‐target fluorescence staining of bacteria smears enables rapid machine learning‐assisted species classification
Source: mLife. 2026 Apr 16;5(2):229–38. doi: 10.1002/mlf2.70076 (PMC13131325; doi:10.1002/mlf2.70076)
Supplement: Supplementary file 1 — suppl material revised‐2. [file MLF2-5-229-s001.docx]

**Supplemental material**

to

**Multi-target fluorescence staining of bacteria smears enables rapid machine learning-assisted species classification**

by

Maxence Galvan, Michael Fujarski, Can Beslendi, Frieder Schaumburg, Julian Varghese, Johannes Liesche

**Content:**

Tables S1, S2

Figures S1 to S9

**Supplied as separate file:** Table S2

**Supplemental tables**

Table S1: Information on fluorescent dyes used in this study.

| **Dye** | **Biological Target(s)** | **Solvent** | **Stock/**  **Working Concentration** | **Filter set: Excitation filter**  **Dichroic mirror**  **Emission filter** | **Expo-sure time** | **References** |
| --- | --- | --- | --- | --- | --- | --- |
| Acridine Orange | - DNA/ RNA - Acidic vesicles | Water,  Ethanol, DMSO | 0.5 mg/mL | 450 nm - 490 nm  495 nm  500 nm - 550 nm | 100 ms | - Plemel JR, Caprariello AV, Keough MB, et al. Unique spectral signatures of the nucleic acid dye acridine orange can distinguish cell death by apoptosis and necroptosis. *J Cell Biol*. 2017;216(4):1163-1181. Pierzyńska-Mach A, Janowski PA, Dobrucki JW. Evaluation of acridine orange, LysoTracker Red, and quinacrine as fluorescent probes for long-term tracking of acidic vesicles. *Cytometry A*. 2014;85(8):729-737. |
| Auramine O | - Mycolic acids in cell walls - Protein aggregates | Water, Ethanol | 1 mg/mL  100 µg/mL | 450 nm - 490 nm  495 nm  500 nm - 550 nm | 50 ms | - Vilchèze C, Kremer L. Acid-Fast Positive and Acid-Fast Negative Mycobacterium tuberculosis: The Koch Paradox. *Microbiol Spectr*. 2017;5(2):10.1128/microbiolspec.tbtb2-0003-2015. - Mudliar NH, Sadhu B, Pettiwala AM, Singh PK. Evaluation of an Ultrafast Molecular Rotor, Auramine O, as a Fluorescent Amyloid Marker. *J Phys Chem B*. 2016;120(40):10496-10507. |
| Calcofluor White | - Chitin - Cellulose - β-1,3-glucans | Water | 1 mg/mL  (stock = working solution) | < 365 nm  395 nm  420 nm - 470 nm | 100 ms | - Haigler CH, Brown RM Jr, Benziman M. Calcofluor white ST Alters the in vivo assembly of cellulose microfibrils. *Science*. 1980;210(4472):903-906. - García-Rodriguez LJ, Durán A, Roncero C. Calcofluor antifungal action depends on chitin and a functional high-osmolarity glycerol response (HOG) pathway: evidence for a physiological role of the Saccharomyces cerevisiae HOG pathway under noninducing conditions. *J Bacteriol*. 2000;182(9):2428-2437. Rasconi S, Jobard M, Jouve L, Sime-Ngando T. Use of calcofluor white for detection, identification, and quantification of phytoplanktonic fungal parasites. *Appl Environ Microbiol*. 2009;75(8):2545-2553. |
| Congo Red | - Cross-β amyloid fibrils - Cellulose - Chitin - β-1,3-glucans | Water, Ethanol | 1 mg/mL  100 µg/mL | 538 nm - 563 nm  570 nm  570 nm - 640 nm | 300 ms | - Khurana R, Uversky VN, Nielsen L, Fink AL. Is Congo red an amyloid-specific dye?. *J Biol Chem*. 2001;276(25):22715-22721. - Reichhardt C, McCrate OA, Zhou X, Lee J, Thongsomboon W, Cegelski L. Influence of the amyloid dye Congo red on curli, cellulose, and the extracellular matrix in E. coli during growth and matrix purification. *Anal Bioanal Chem*. 2016;408(27):7709-7717. - Roncero C, Durán A. Effect of Calcofluor white and Congo red on fungal cell wall morphogenesis: in vivo activation of chitin polymerization. *J Bacteriol*. 1985;163(3):1180-1185. - Kopecká M, Gabriel M. The influence of congo red on the cell wall and (1----3)-beta-D-glucan microfibril biogenesis in Saccharomyces cerevisiae. *Arch Microbiol*. 1992;158(2):115-126. |
| Propidium Iodide | - DNA, RNA - Pectin (homogalacturonan) | Water,  Ethanol, DMSO | 100 µg/mL  10 µg/mL | 538 nm - 563 nm  570 nm  570 nm - 640 nm | 50 ms | - Cui HH, Valdez JG, Steinkamp JA, Crissman HA. Fluorescence lifetime-based discrimination and quantification of cellular DNA and RNA with phase-sensitive flow cytometry. *Cytometry A*. 2003;52(1):46-55. - Rosenberg M, Azevedo NF, Ivask A. Propidium iodide staining underestimates viability of adherent bacterial cells. *Sci Rep*. 2019;9(1):6483. Published 2019 Apr 24. - Rounds CM, Lubeck E, Hepler PK, Winship LJ. Propidium iodide competes with Ca(2+) to label pectin in pollen tubes and Arabidopsis root hairs. *Plant Physiol*. 2011;157(1):175-187. |
| Rhodamine B | - DNA - Keratin - Membrane potential (mitochondria) - Lysosomes and other acidic compartments | Water, Ethanol, DMSO | 1 mg/mL  100 µg/mL | 538 nm - 563 nm  570 nm  570 nm - 640 nm | 10 ms | - Liisberg MF. Rhodamine B as an extremely specific stain for cornification. *Acta Anat (Basel)*. 1968;69(1):52-57. - Reungpatthanaphong P, Dechsupa S, Meesungnoen J, Loetchutinat C, Mankhetkorn S. Rhodamine B as a mitochondrial probe for measurement and monitoring of mitochondrial membrane potential in drug-sensitive and -resistant cells. *J Biochem Biophys Methods*. 2003;57(1):1-16. - Islam MM, Chakraborty M, Pandya P, Al Masum A, Gupta N, Mukhopadhyay S. Binding of DNA with Rhodamine B: spectroscopic and molecular modeling studies. Dyes Pigments. 2013;99(2):412-422. Minier C, Moore M. Rhodamine B accumulation and MXR protein expression in mussel blood cells—effect of exposure to vincristine. Mar Ecol Prog Ser. 1996;142:165-173. |
| Rose Bengal | - Glycoproteins - Positively charged proteins and lipids | Water,  DMSO | 1 mg/mL  100 µg/mL | 538 nm - 563 nm  570 nm  570 nm - 640 nm | 50 ms | - Feenstra RP, Tseng SC. What is actually stained by rose bengal?. *Arch Ophthalmol*. 1992;110(7):984-993. - Doughty MJ. Rose bengal staining as an assessment of ocular surface damage and recovery in dry eye disease-a review. *Cont Lens Anterior Eye*. 2013;36(6):272-280. |
| Trypan Blue | - Proteins - Cell walls | Water | 1 mg/mL  100 µg/mL | 538 nm - 563 nm  570 nm  570 nm - 640 nm | 200 ms | - Lang JH, Lasser EC. Spectrophotometric studies of the binding of trypan blue to bovine serum albumin. *Biochemistry*. 1967;6(8):2403-2409. Liesche J, Marek M, Günther-Pomorski T. Cell wall staining with Trypan blue enables quantitative analysis of morphological changes in yeast cells. *Front Microbiol*. 2015;6:107. Published 2015 Feb 11. |

Table S2: Raw feature data used for training and testing the machine learning model. [supplied as separate file]

**Supplemental figures**

**Figure S1**


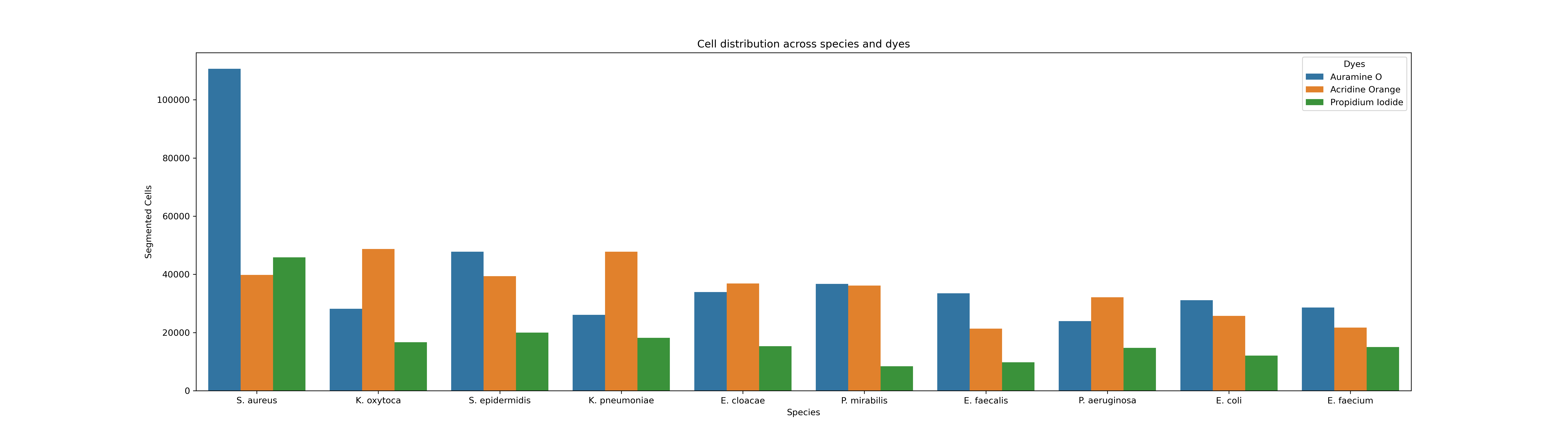


Fig. S1: Composition of the test-train-dataset with regard to the number of cells analyzed per dye per species.

**Figure S2**

**
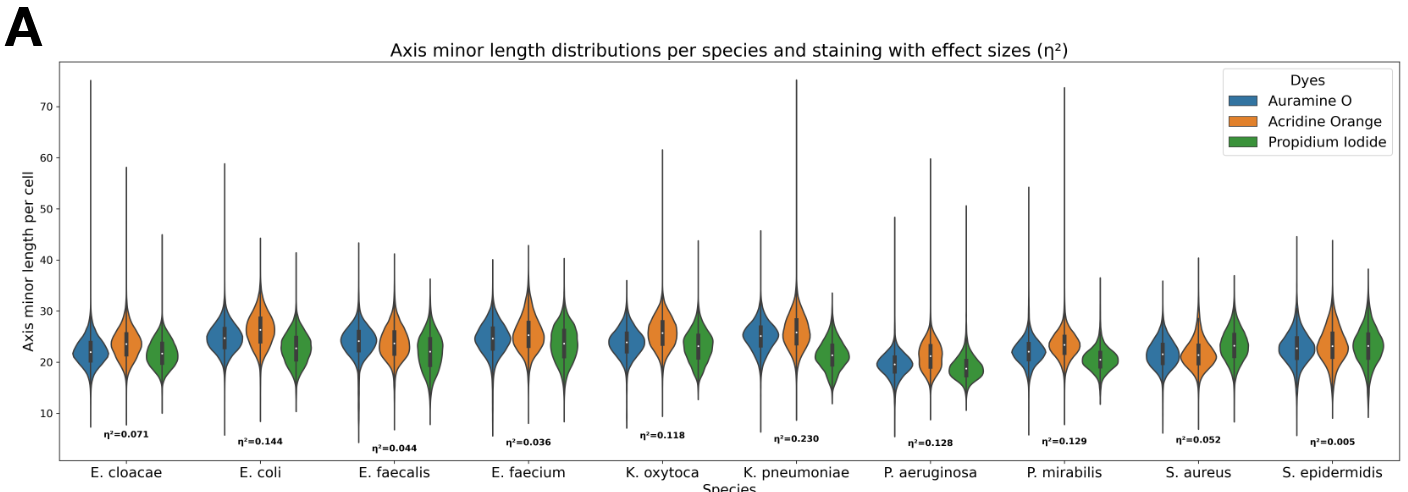
**

**
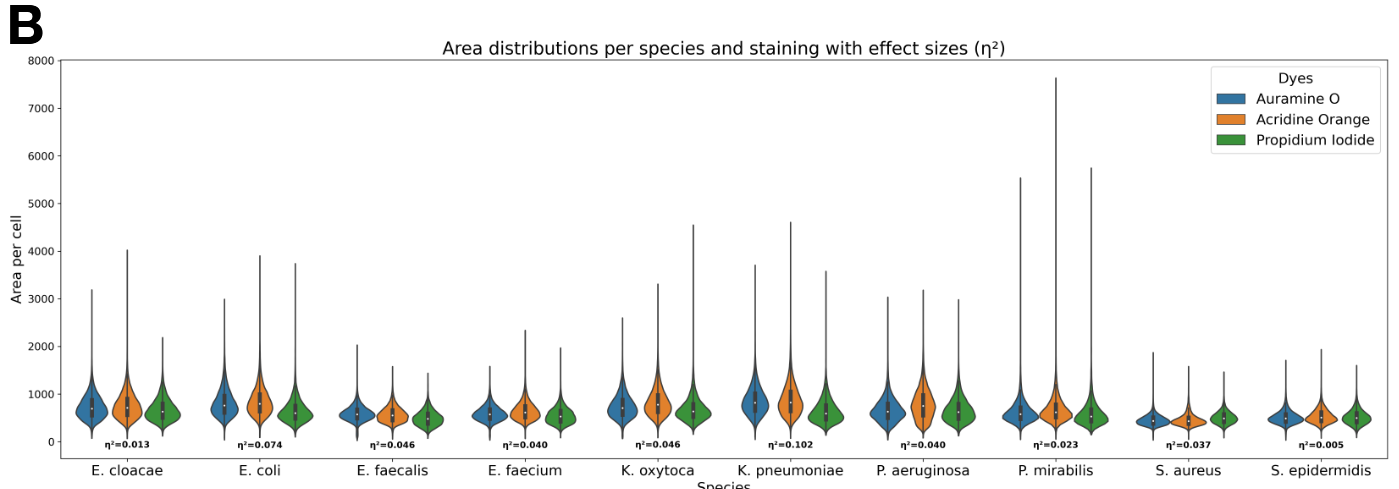
**

**
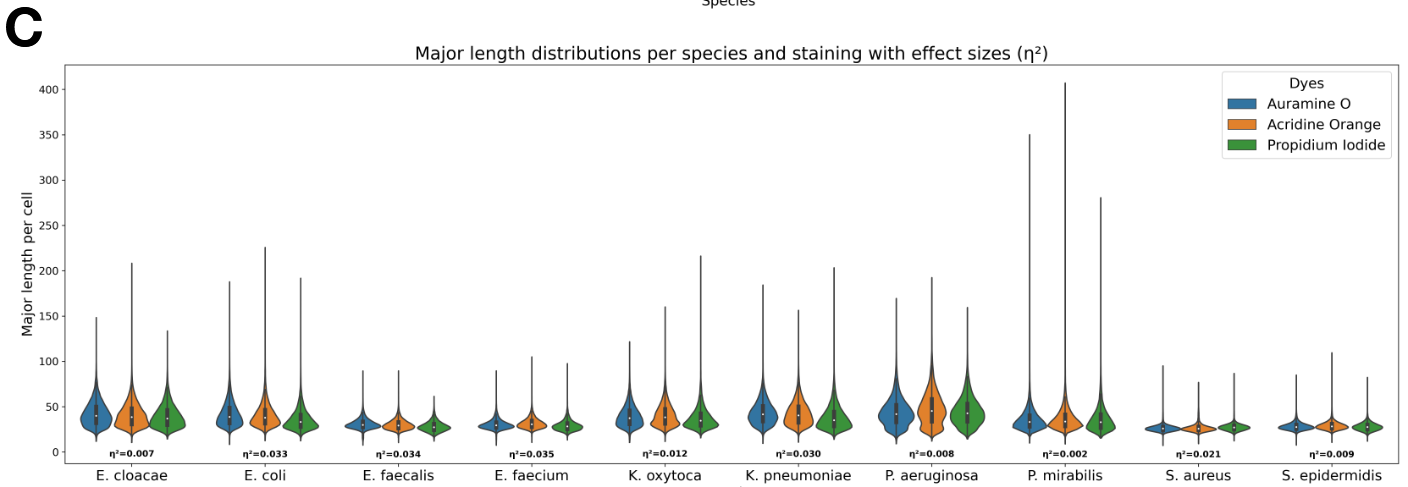
**


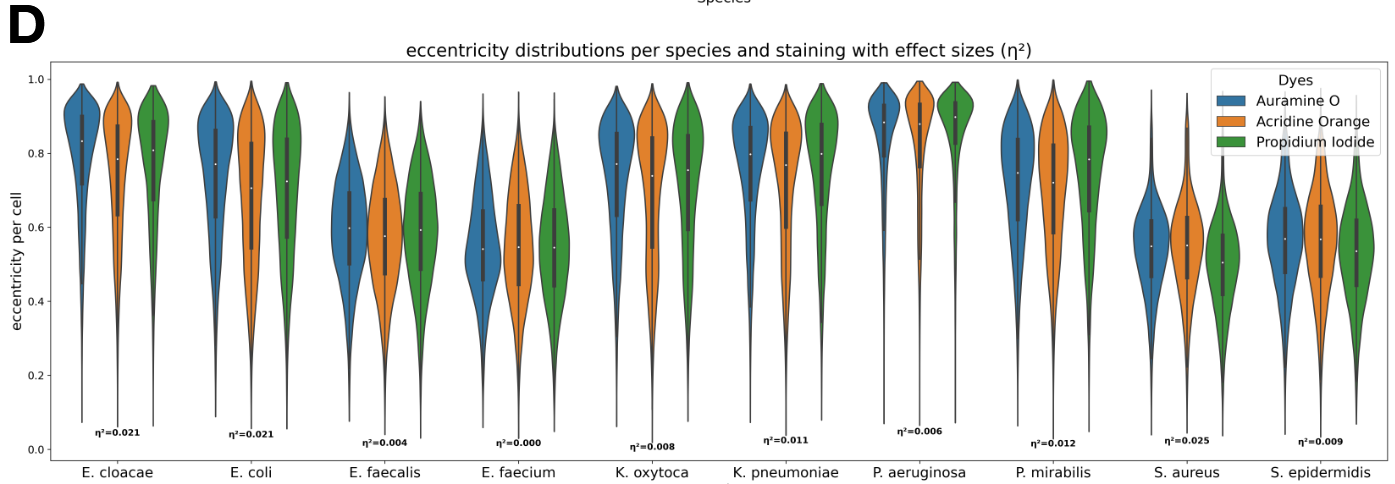


Fig. S2: Violin plots of microbial structural parameters across species and staining conditions. (A) Minor axis length, (B) cell area, (C) major axis length, and (D) eccentricity are shown for each species and dye (Auramine O, Acridine Orange, Propidium Iodide). Effect sizes (η²) are reported for each species.

**Figure S3**


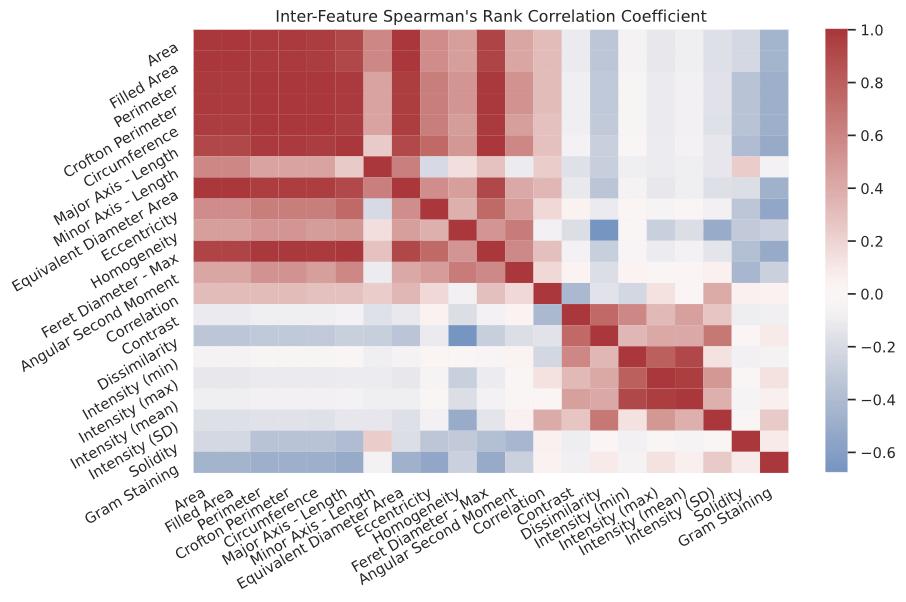


Fig. S3: Correlation analysis of cell shape- and staining-related features. Colors indicate coefficients of spearman-rank correlation. N > 800,000.

**Figure S4**


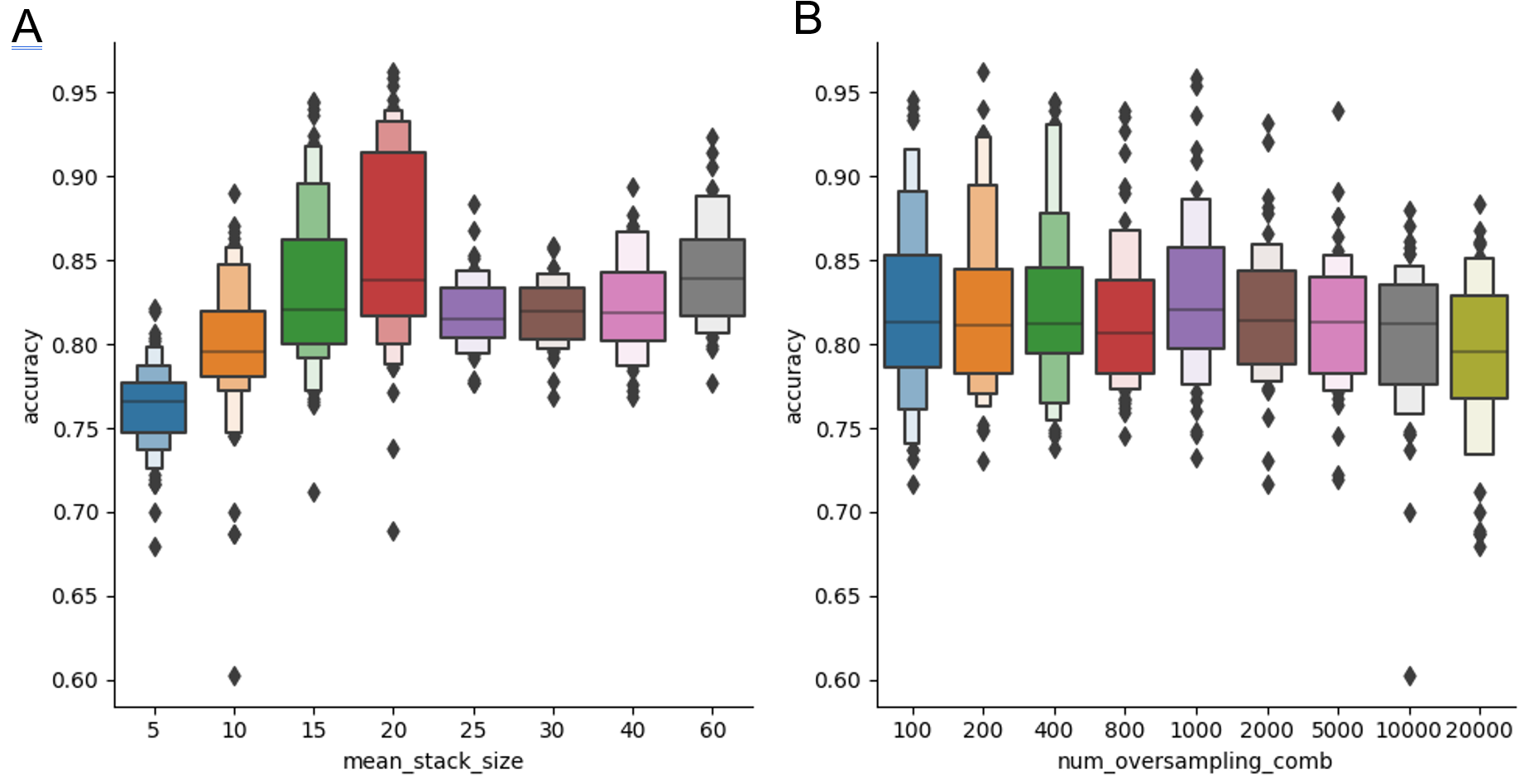


Fig. S4: Impact of Mean Stack Size (A) and Oversampling Combinations (B) on Model Accuracy.

**Figure S5**

**
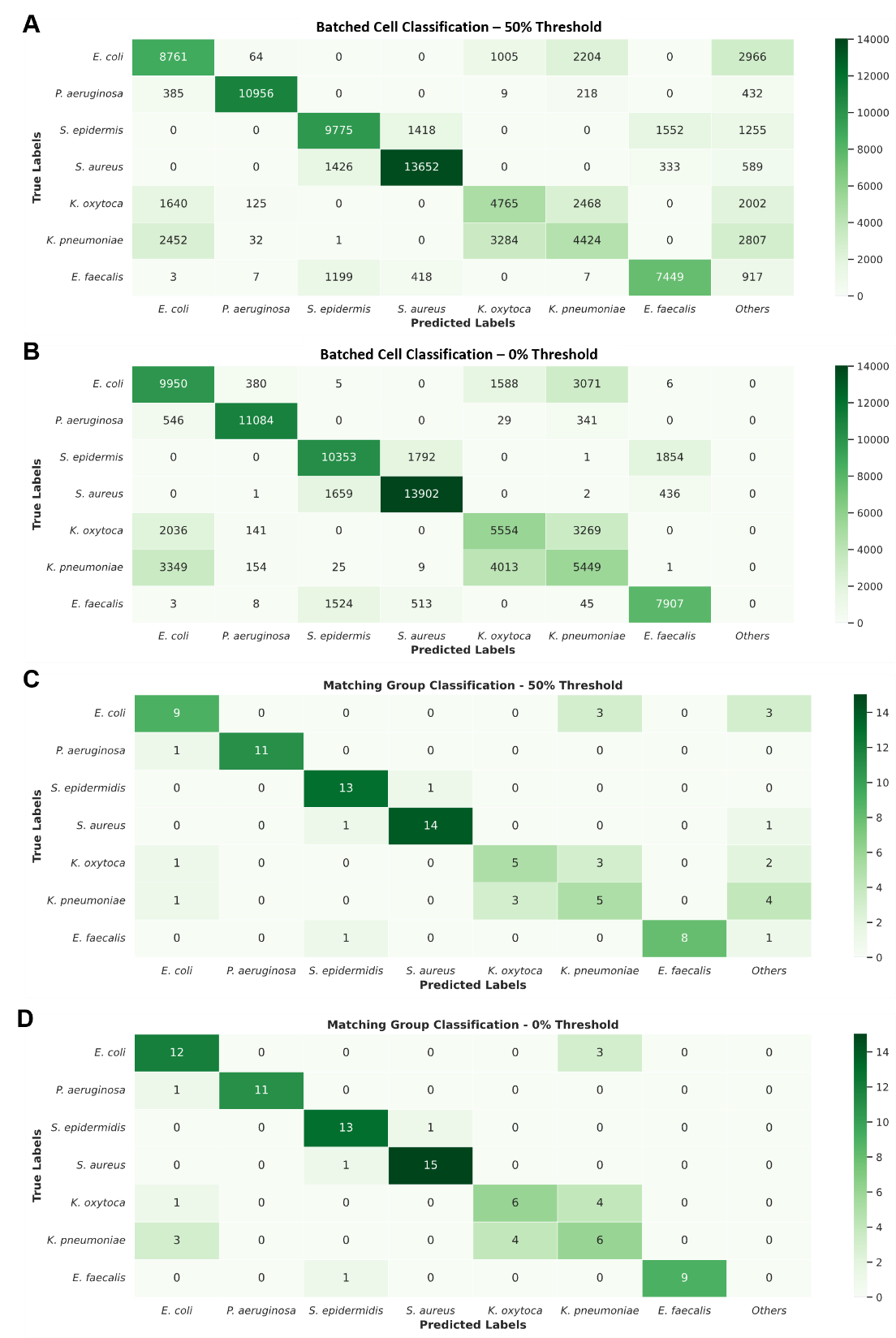
**

**Fig. S5: Influence of prediction accuracy threshold on classification of species included in the model training.** Confusion matrices of the classification model, either processing batched cell data (**A,B**) or majority votes on 1000 batched cell sample sets (**C,D**) indicate the number of correctly classified and misclassified samples. “Others” refers to instances which could not be classified as one of the species included in the training set with a prediction accuracy above 50% (A,C) or 0% (B,D).

**Figure S6**


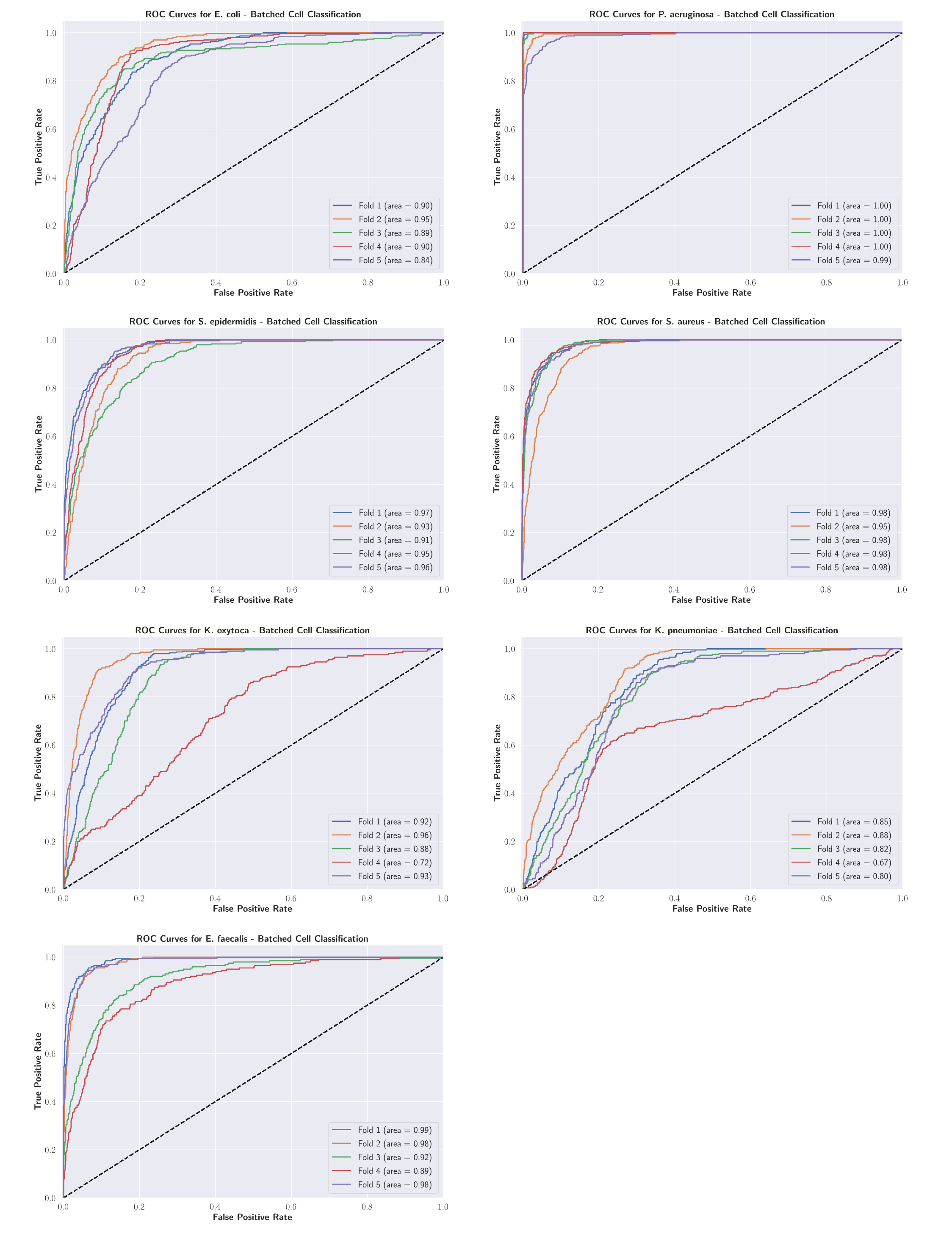


Fig. S6: Receiver operating characteristic (ROC)-curves indicating the performance of the batched cell classification for the different bacteria as the area under the curve for the different folds of cross-validation.

**Figure S7**


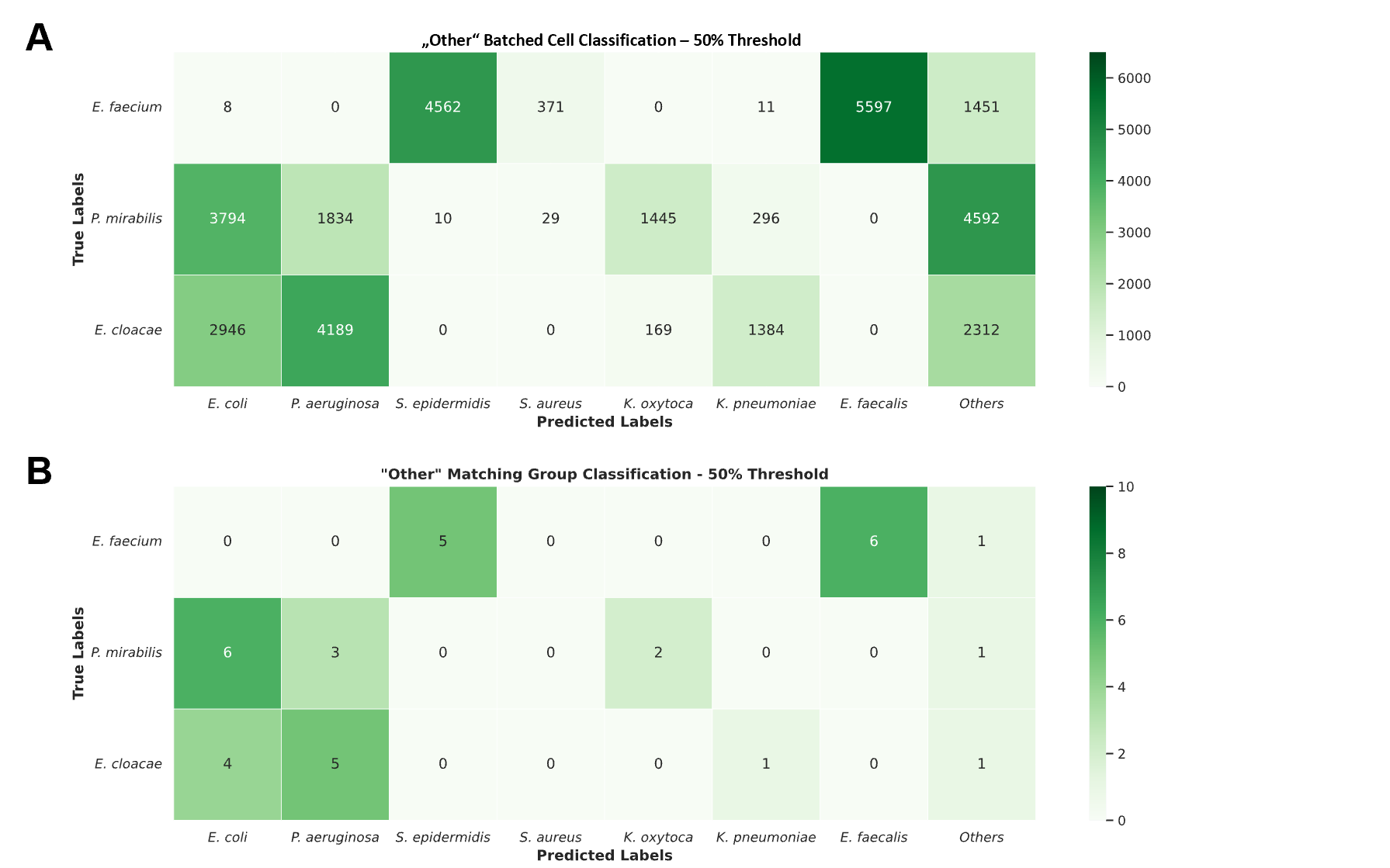


Fig. S7: Influence of prediction accuracy threshold on classification of species not included in model training. Confusion matrices of the classification model, either processing batched cell data (**A**) or majority votes on 1000 batched cell sample sets (**B**) indicate the number of correctly classified and misclassified samples. “Others” refers to instances which could not be classified as one of the species included in the training set with a prediction accuracy above 50%.

**Figure S8**


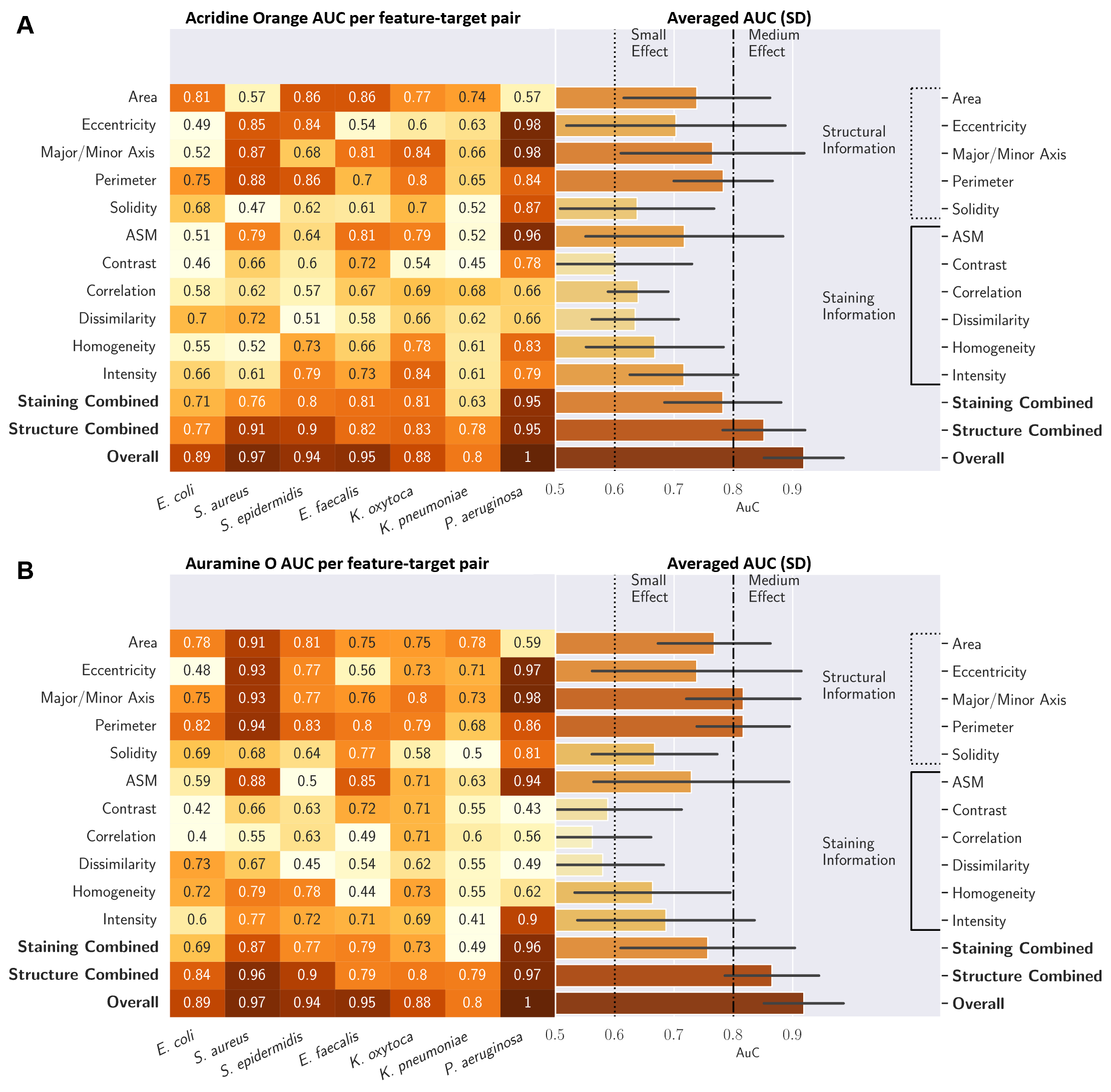


Fig. S8: Feature importance analysis for classification performance of individual dyes: Acridine Orange (A) and Auramine O (B). The effect of the permutation of individual features on model performance was tested. Conventional effect thresholds at 0.6 (small effect) and 0.8 (medium effect) were used to evaluate the importance of individual features according to the respective area under the curve (AUC) of the receiver-operating characteristics. Error bars show standard deviation (SD).

**Figure S9**


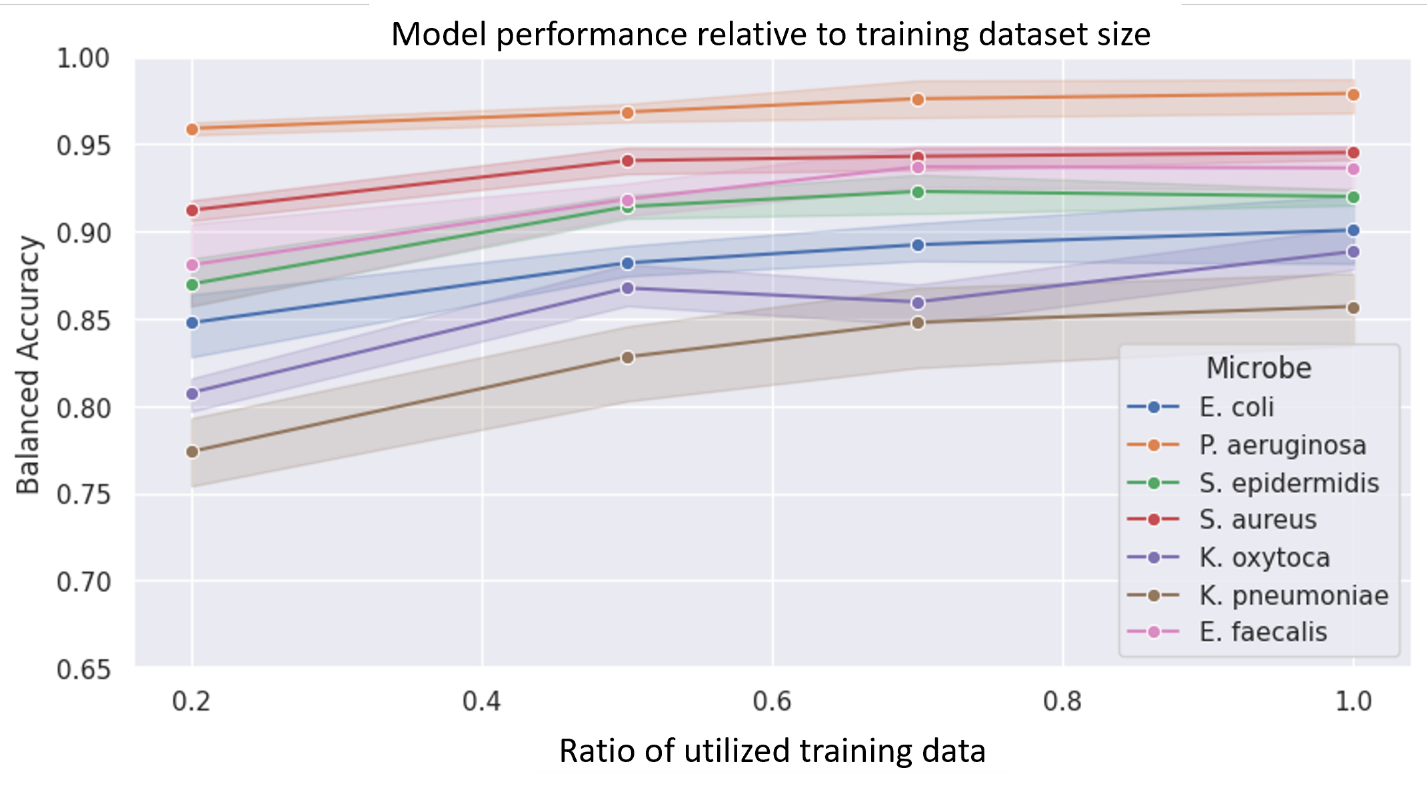


Fig. S9: Comparison of model performance per microbe for different ratios of utilized training data. The amount of utilized data was evaluated by subsetting it to 20%, 50%, and 70%, and comparing it to the original 100% of training data. The size of the test dataset, used for evaluation, was constant. Reducing the training data resulted in underfitting of the models.
